# *Sporothrix brasiliensis* Treatment Failure without Initial Elevated Itraconazole MICs in Felids at Border of Brazil

**DOI:** 10.3201/eid3109.250156

**Published:** 2025-09

**Authors:** Carolina Melchior do Prado, Bram Spruijtenburg, Emanuel Razzolini, Luciana Chiyo, Carlos Santi, Caroline Amaral Martins, Gabriela Santacruz, Nancy Segovia, José Pereira Brunelli, Regielly Caroline Raimundo Cognialli, Jacques F. Meis, Vânia Aparecida Vicente, Theun de Groot, Eelco F.J. Meijer, Flávio Queiroz-Telles

**Affiliations:** Radboudumc–CWZ Center of Expertise for Mycology, Nijmegen, the Netherlands (C.M. do Prado, B. Spruijtenburg, J.F. Meis, T. de Groot, E.F.J. Meijer); Federal University of Paraná, Curitiba, Brazil (C.M. do Prado, E. Razzolini, R.C.R. Cognialli, V.A. Vicente, F. Queiroz-Telles); Canisius-Wilhelmina Hospital (CWZ)/Dicoon, Nijmegen (B. Spruijtenburg, T. de Groot, E.F.J. Meijer); Zoonosis Control Center, Foz do Iguaçu, Brazil (L. Chiyo, C. Santi, C.A. Martins); National University of the East, Minga Guazú, Paraguay (G. Santacruz, N. Segovia); Ministry of Public Health and Social Welfare, Asuncion, Paraguay (J.P. Brunelli); Institute of Translational Research, Cologne Excellence Cluster on Cellular Stress Responses in Aging-Associated Diseases, Excellence Center for Medical Mycology, University of Cologne, Cologne, Germany (J.F. Meis)

**Keywords:** Sporothrix brasiliensis, fungi, zoonoses, felids, feline, cats, antimicrobial resistance, fungi, zoonoses, antifungal susceptibility testing, short tandem repeats, genotyping, sporotrichosis, zoonotic transmission, epidemiology, antifungal resistance, itraconazole, triple border region, Triple Frontier, Argentina, Paraguay, Brazil

## Abstract

Cat-transmitted sporotrichosis caused by *Sporothrix brasiliensis* is an emerging zoonosis in Latin America. Because treatment of feline sporotrichosis is often not effective, we sought to determine whether treatment failure results from *S. brasiliensis* strains that have existing elevated MICs for itraconazole, the primary treatment for this disease. During 2021–2023 at the triple border region of Brazil, Paraguay, and Argentina, 108 *S. brasiliensis* strains were isolated from felines before antifungal treatment. The main clinical manifestation was cutaneous disseminated sporotrichosis (61%), which was the only form resulting in sporotrichosis-induced deaths (61%). We conducted antifungal susceptibility testing for 9 antifungal compounds, evaluating for both mycelial and yeast phases. MIC levels were low for most antifungal agents but were higher in the mycelial phase than in the yeast phase, especially for voriconazole and isavuconazole. We conclude that the varying clinical manifestations of sporotrichosis and large differences in mortality rates were not caused by elevated itraconazole MICs.

Sporotrichosis is a globally neglected epizoonotic and sapronotic disease, primarily affecting the skin and subcutaneous tissues, caused by fungi of the *Sporothrix* genus, and represents the most prevalent implantation mycosis in Latin America, especially in Brazil (*1*). *Sporothrix* spp. are thermally dimorphic fungi from the order Ophiostomatales, showing filamentous forms at 25°C–30°C in the environment and yeast-like forms at temperatures of 35°C–37°C, as in mammals (*2*). The main clinical pathogenic species are *S. brasiliensis*, *S. schenckii*, *S. globosa*, and *S. luriei*. *S. schenckii* and *S. globosa* are usually transmitted via the sapronotic route, involving traumatic implantation with plant or soil debris (*3*). During the past 3 decades, zoonotic transmission of *S. brasiliensis* from infected cats to humans, other felids, and canines has resulted in multiple outbreaks in Brazil and other Latin America countries (*4*–*7*). Cat-transmitted sporotrichosis caused by *S. brasiliensis* is a major public health concern in Latin America. Infections are rapidly spreading from Brazil to other countries, and cases have been described in Brazil (*8*,*9*), Argentina (*5*,*10*), Paraguay (*4*), and Chile (*6*). In addition, imported cases in the United Kingdom (*11*) and United States (*12*) have been reported. Transmission by infected cats, via yeast form (*13*), occurs through bites, scratches, direct contact with exudate from skin lesions, and probably through respiratory droplets by cat sneezes (*2*,*14*). Cats are the primary animal hosts and main source of infection for other cats, dogs, and humans (*15*).

Cat-transmitted sporotrichosis outbreaks often involve clonal zoonotic transmission (*8*,*16*). To curb such outbreaks, one of the necessary measures is antifungal treatment of cats (*15*). The drug of choice is itraconazole, although various refractory cases have been reported (*17*,*18*). Whether treatment failure results from high antifungal MICs is unknown because that possibility has been poorly investigated (*19*). Recently, high antifungal MICs against itraconazole were reported in isolates obtained from both cats and humans (*19*–*21*). Whether strains with reduced susceptibility are also transmitted or whether reduced susceptibility only develops during treatment remain unclear. Antifungal susceptibility testing (AFST), applicable to both the yeast and mycelial form, has not been standardized in dimorphic fungi, leading to different protocols. As a consequence, published MICs are currently difficult to compare. 

We investigated the spread of feline sporotrichosis in the triple border region between Brazil, Paraguay, and Argentina by molecular genotyping. In addition, we obtained MICs for common antifungal drugs using microbroth dilution methods of both the yeast and mycelial phase to determine whether cats with sporotrichosis had *S. brasiliensis* with elevated itraconazole MICs at the onset of treatment. This study was approved by the Committee for Ethics in Research of the Federal University of Paraná (approval no. CAAE 52726021.8.0000.0102) and by the Animal Use Ethics Committee of the Federal University of Paraná, Curitiba, Brazil.

## Materials and Methods

### Isolate and Data Collection

During July 2021–October 2023, we collected swab samples from 108 symptomatic cats that had lesions compatible with sporotrichosis. All cats lived in the triple border region between Brazil, Paraguay, and Argentina. Cats were selected through notification of the owners; health agents of the Zoonosis Control Center of Foz do Iguaçu, Brazil; veterinarians from private clinics and hospitals; and receipt at the Zoonosis Control Center of animals suspected to be infected. In Foz do Iguaçu, samples were collected at the homes of the cats or at the place indicated by the citizen in cases of stray cats. In Paraguay, samples were collected at private veterinary clinics. We obtained clinical and environmental data by using questionnaires sent to the owners of each cat. 

We evaluated and classified all cats according to the types of their lesions and divided them into 3 groups: cutaneous disseminated, cats with ulcerated lesions in different parts of the body and systemic signs; fixed cutaneous, cats with single ulcerated lesion without systemic signs; and extracutaneous, cats without ulcerated lesions but with other clinical manifestations including sneezing, dyspnea, nasal discharge, and other respiratory symptoms. We used cartographic bases from the Brazilian Institute of Geography and Statistics (IBGE) for georeferencing the coordinates where cats lived and QGIS software (https://qgis.org) to assemble the maps. To determine clinical outcomes, we followed the cats for the duration of treatment, until they died, recovered, or were lost to follow-up.

### Diagnosis and Molecular Investigation

We diagnosed sporotrichosis via fungal culture of swab specimens collected from the wounds, as previously described (*4*). We cultivated specimens on Sabouraud dextrose agar (KASVI, https://www.kasvi.com.br) containing chloramphenicol and incubated at 25°C–27°C for up to 10 days. We performed micromorphology of colonies to confirm *Sporothrix* growth and calmodulin sequencing for species identification, as previously described (*4*). As control isolates, we used *S. brasiliensis* CBS 133017 (GenBank accession no. KP101458.1), *S. schenckii* CBS 117440 (accession no. KP101386.1), *S. globosa* CBS 129721 (accession no. KP101478.1), *S. luriei* ATCC 18616 (accession no. KT427639.1), *S. mexicana* Ss133 (accession no. JF811341.1), *S. chilensis* Ss470 (accession no. KP711816.1), *S. humicola* CBS 118129 (accession no. KX590808.1), and *S. phasma* CBS 119721 (accession no. KX590795.1). We deposited sequences generated in this study into GenBank (accession nos. OR501574, OR501573, and PQ741608–713) (Appendix Table 1). We performed genotyping of isolates using short tandem repeats, as previously described (*16*) (Appendix).

### AFST

We performed AFST for the mycelial and yeast phases of all isolates using broth microdilution as outlined in Clinical and Laboratory Standards Institute (CLSI) reference standard M38 for the mycelial phase (*22*,*23*) and CLSI reference standard M27 for the yeast phase (*24*) (Appendix). For the mycelial phase, we cultured isolates on potato dextrose agar (Sigma Aldrich, https://www.sigmaaldrich.com) plates at 30°C for 7 days and confirmed the absence of yeast cells microscopically. For the yeast phase, we cultured isolates onto brain–heart infusion plates (Xebios Diagnostics GmbH, https://www.xebios.com) at 35°C for 7 days, then performed a second passage on brain–heart infusion plates at 35°C for 7 days. We then microscopically confirmed the absence of filamentous fungi. 

## Results

### Clinical Epidemiology

**Figure 1:**
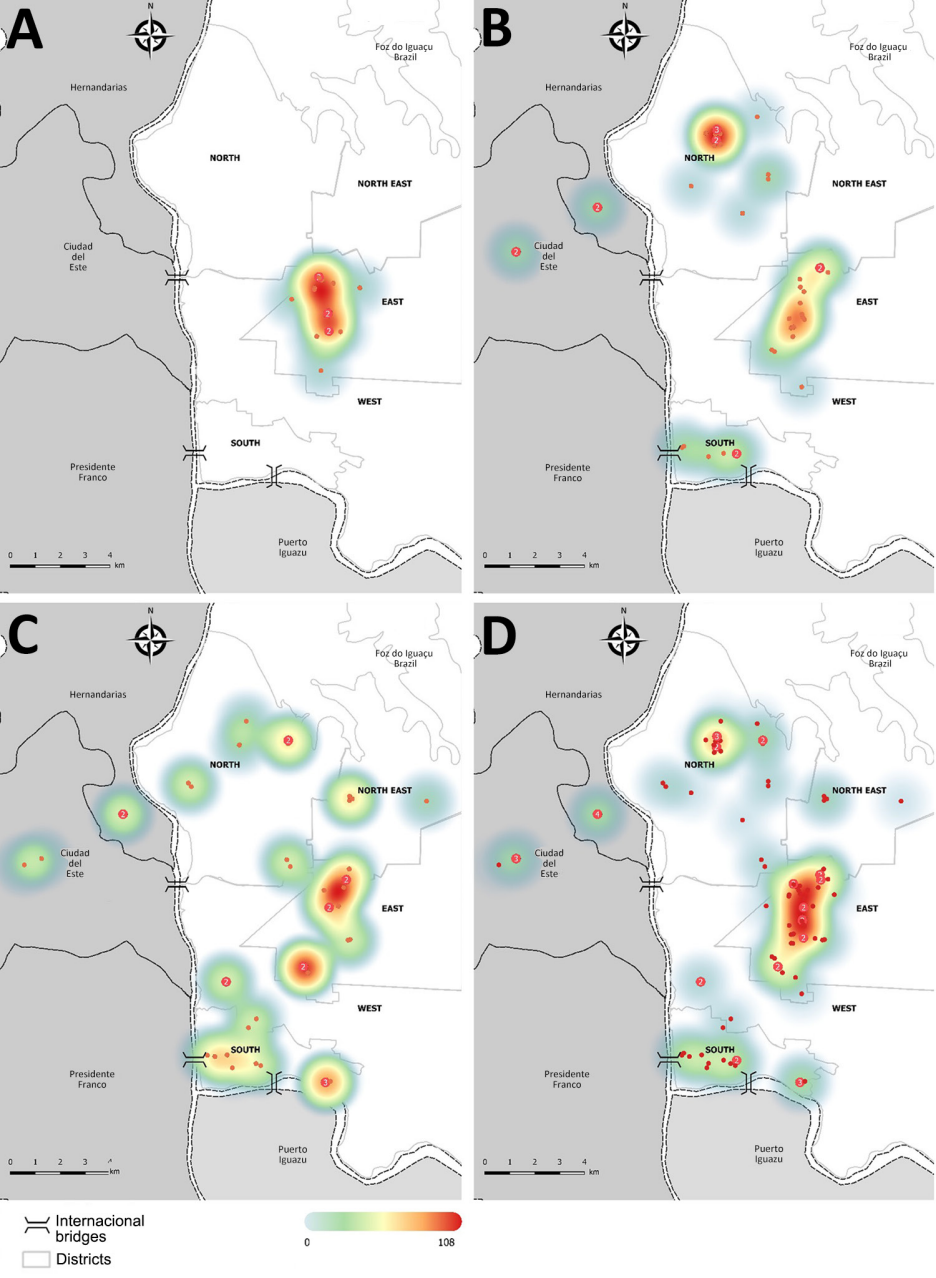
Heatmaps showing the spatial distribution of cats with proven sporotrichosis in the triple border region of Brazil (white), Paraguay (dark gray), and Argentina (light gray) during 2021–2023 in study of *Sporothrix brasiliensis* treatment failure without initial elevated itraconazole MICs in felids at border of Brazil. Red dots show locations of feline sporotrichosis cases during 2021 (A), 2022 (B), and 2023 (C) and for all 3 years combined (D). Numbers in red dots indicate multiple positive cats in the same house.

We obtained 108 isolates of *Sporothrix* spp. originating from 88 households (Appendix Table 1). Of the animals, 100 were from Foz do Iguaçu (Brazil), 4 from Ciudad del Este (Paraguay), and 4 from Hernandarias (Paraguay). No animals had a history of travel to other regions. Sporotrichosis cases were initially only found in neighborhoods in the eastern region of Foz do Iguaçu (Figure 1, panel A), but over time, cases were found in other regions, close to the country border, especially around the international bridges (Figure 1, panels B, C). The prevalence of sporotrichosis cases was highest in the northern, southern, and eastern districts (Table 1), regions with the highest density of humans (Appendix Figure 1). Furthermore, most cases were found at or close to low-income urban communities and favelas (Appendix Figure 2), which are low-income, dense housing settlements, characterized by low socioeconomic status, precarious conditions, and lack of essential services, mostly found at the eastern region of Foz do Iguaçu (Appendix Figure 2). Of note, all cats had easy access to the street, other homes, backyards, and vacant lots. Feline sporotrichosis was more frequently in male cats (male:female ratio 2.8:1), adults, uncastrated cats, those not vaccinated for any disease, and those with little or no access to veterinary services; most cats did have an owner (Table 2). 

**Table 1:** Prevalence of *Sporothrix brasiliensis* among cats in study of treatment failure without initial elevated itraconazole MICs in felids at border of Brazil

District	No. residences	No. positive animals	Prevalence/1,000 residences
North	32,610	24	0.73
West	32,705	3	0.09
South	16,175	17	1.05
North East	18,804	4	0.21
East	36,315	52	1.43

**Table 2:** Characteristics of cats tested in study of *Sporothrix brasiliensis* treatment failure without initial elevated itraconazole MICs in felids at border of Brazil

Characteristic	No. (%) cats	
	Sporotrichosis	No sporotrichosis
Total no.	108	25
Sex		
M	80 (74)	17 (68)
F	28 (26)	8 (32)
Age, mo.		
<12	13 (12)	3 (12)
>12	95 (88)	22 (88)
Castration status		
Uncastrated	81 (75)	12 (48)
Castrated	23 (21)	12 (48)
Unknown	4 (4)	1 (4)
Vaccination status		
Never vaccinated	64 (59)	14 (56)
Full vaccination	11 (10)	4 (16)
Only primary	12 (11)	3 (12)
Occasional	4 (4)	3 (12)
Unknown	17 (16)	1 (4)
Access to veterinary care		
No access	76 (70)	22 (88)
With access	32 (30)	3 (12)
Ownership		
With an owner	95 (88)	24 (96)
Stray cat	13 (12)	1 (4)

The main clinical manifestation was cutaneous disseminated sporotrichosis (61%), followed by fixed cutaneous (34%) and extracutaneous (5%) sporotrichosis (Table 3). The mortality rate was 61% for cats with the disseminated form, but no cats with the fixed cutaneous or extracutaneous forms died from sporotrichosis. Ten animals were euthanized because they lived on the street without owners, hindering any possibility of treatment; 8 cats were lost to follow-up.

**Table 3:** Outcomes of cats with sporotrichosis according to clinical form of disease in study of *Sporothrix brasiliensis* treatment failure without initial elevated itraconazole MICs in felids at border of Brazil*

Outcomes	No. (%) cats
Cutaneous disseminated disease, n = 66	
Death	40 (61)
Clinical cure	16 (24)
Lost to follow-up	3 (4)
Euthanized	7 (11)
Fixed cutaneous disease, n = 37	
Clinical cure	29 (78)
Lost to follow-up	5 (14)
Euthanized	3 (8)
Extracutaneous disease, n = 5†	
Clinical cure	5 (100)

*Total number of cats with sporotrichosis was 108. †Absence of skin lesions but presence of extracutaneous signs, such as sneezing, dyspnea, and nasal discharge and other respiratory signs.

All cats received itraconazole (25–100 mg/d), and those with disseminated and extracutaneous forms also received potassium iodide (2.5–20 mg/kg/24 h, depending on the severity of symptoms). For the clinically cured cats, treatment duration ranged from 2 to 15 months, and we observed no correlation to the clinical form (Table 4). For the cats that died, 12 never received treatment; for the others, treatment duration ranged from 1 week to 7 months.

**Table 4:** Treatment regimen and duration among 50 clinically cured cats and 40 that died from sporotrichosis according to clinical form of disease in study of *Sporothrix brasiliensis* treatment failure without initial elevated itraconazole MICs in felids at border of Brazil

Sporotrichosis treatment	Duration	No. cats	
		Clinically cured	Died
Cutaneous disseminated disease, n = 56			
No treatment applied	Not applicable	0	12
Itraconazole + potassium iodide	1–4 wk	0	12
Itraconazole + potassium iodide	2–7 mo	3	16
Itraconazole + potassium iodide	8–12 mo	11	0
Itraconazole + potassium iodide	13–15 mo	2	0
Fixed cutaneous disease, n = 29			
Itraconazole	2–6 mo	12	0
Itraconazole	7–12 mo	17	0
Extracutaneous disease, n = 5			
Itraconazole + potassium iodide	2–6 mo	5	0

### Phylogenetic Analysis

**Figure 2:**
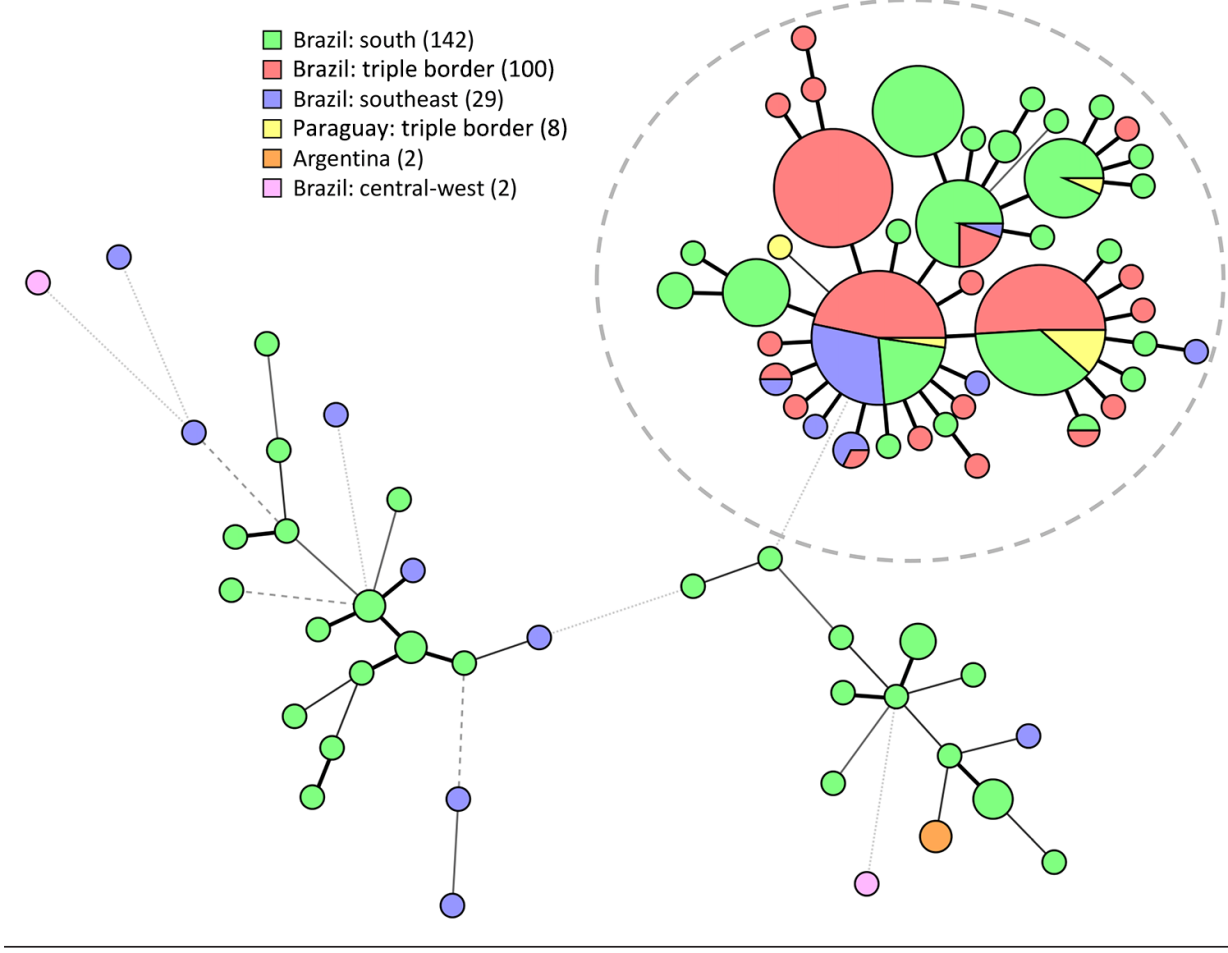
Minimum-spanning tree of isolates in study of *Sporothrix brasiliensis* treatment failure without initial elevated itraconazole MICs in felids at border of Brazil. Tree comprises 283 isolates, including 108 isolates based on 9 short tandem repeat markers from this study (red and yellow); green, blue, and pink indicate comparison isolates from previous studies; and numbers in the key represent the number of isolates from each location. Gray dashed circle indicates Rio de Janeiro clade. Regions of Brazil: south, Paraná, Rio Grande do Sul; southeast, Rio de Janeiro, Minas Gerais, São Paulo, Espírito Santo; central-west, Federal District.

Calmodulin sequencing identified all 104 isolates as *S. brasiliensis*, displaying no genetic variation within that gene. Short tandem repeat genotyping yielded 20 genotypes, of which 6 previously had been found in other regions (*16*) (Figure 2). All isolates from the triple border region were highly related, and all grouped within the Rio de Janeiro clade, a previously described dominant group of genotypes originating from Rio de Janeiro, Brazil (*16*). In this study, those isolates often clustered with isolates from other regions of Paraná, Rio de Janeiro, and several other states. In addition, isolates did not cluster based on clinical outcome; all clusters contained isolates from cats who were clinically cured, died, and were euthanized (Appendix Figure 3).

### MIC Investigation

**Figure 3:**
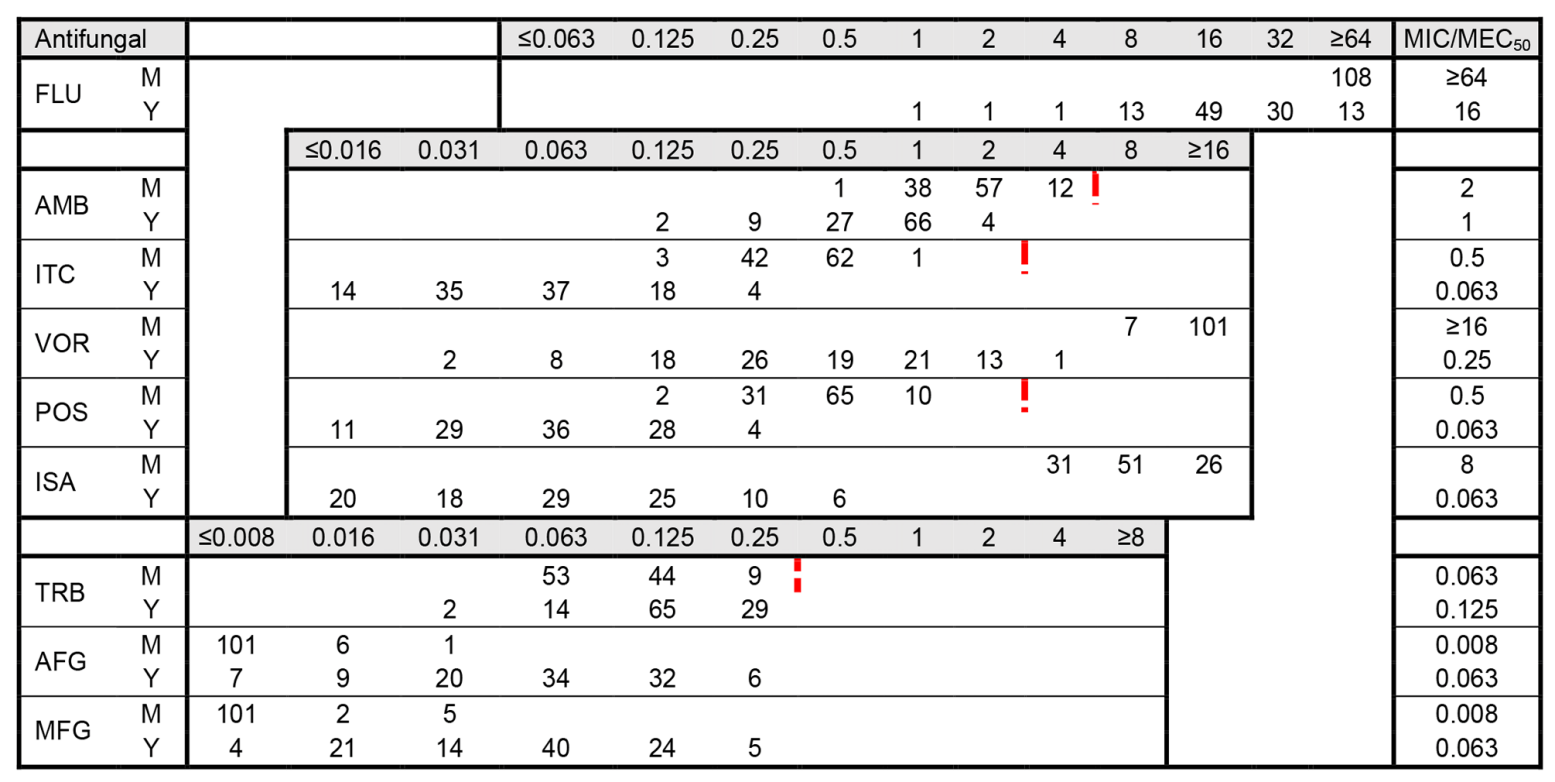
Distribution of MICs for 108 clinical isolates in study of *Sporothrix brasiliensis* treatment failure without initial elevated itraconazole MICs in felids at border of Brazil. MICs were determined according to Clinical Laboratory and Standards Institute M38 (*22*) and M27 (*24*) guidelines. Red dotted lines indicate division of wild-type versus non–wild-type isolates based on ECV values proposed by Espinel-Ingroff (*23*), when available. The ECV value for voriconazole is 32 µg/mL. MICs are given in µg/mL. For AFG and MFG, the MEC_50_ (filamentous phase) was determined. AFG, anidulafungin; AMB, amphotericin B; FLU, fluconazole; ISA, isavuconazole; ITC, itraconazole; M, mycelial phase; MEC_50_, minimal effective concentration that inhibits 50% of isolates; MFG, micafungin; MIC_50_, MIC that inhibits 50% of isolates; POS, posaconazole; TRB, terbinafine; VOR, voriconazole; Y, yeast phase.

For AFST, on the basis of Espinel-Ingroff et al. (*23*), who proposed epidemiological cutoff values (ECVs) based on the M38 CLSI protocol (*22*), we classified all isolates as wild-type for antifungal drugs with available ECVs (Figure 3). For the mycelial phase, itraconazole and posaconazole had the highest in vitro activity, followed by amphotericin B. In contrast, fluconazole, voriconazole, and isavuconazole had low activity and high MICs. For the yeast phase, itraconazole, posaconazole, and isavuconazole showed the highest activity, followed by voriconazole and amphotericin B. Comparing susceptibility levels between both phases, isolates at the mycelial phase had higher MICs for all azoles (for example, differences in 50% MIC values were 8-fold for itraconazole, 64-fold for voriconazole, and 128-fold isavuconazole) and amphotericin B, whereas terbinafine and echinocandins had higher MICs at the yeast phase. Finally, the geometric mean of strains isolated from cats with disseminated disease that recovered was similar to those from cats that died (Appendix Table 2).

## Discussion

Epidemiologic data show that, within 3 years, *S. brasiliensis*–induced sporotrichosis spread across the triple border region of Brazil, Paraguay, and Argentina. The rapidly increasing number of cases in felids highlights the severity of sporotrichosis as a public health problem and the potential for outbreaks (*25*). The data also suggest that cat-transmitted sporotrichosis mainly affects an urban cat population in areas with a high concentration of humans and likely also cats, as compared with areas with a low density of humans (*8*,*26*). All cats in this study were free to roam outside with access to the street, other homes, backyards, and vacant lots, and although no cats had reported travel history, they were also able to roam freely across national borders in this region. We observed introduction of sporotrichosis into Paraguay near the international bridges in the area. Furthermore, based on general assumptions, cases correlate with low socioeconomic status; the eastern region of Foz do Iguaçu has a low overall standard of living. As a consequence, most cats had owners but no access to a veterinarian (*27*). In most cases, owners were not financially able to provide basic resources for their cats’ health and, in cases of sporotrichosis, they were not able to provide diagnosis and treatment. Therefore, public policies that provide such tools free of charge are crucial.

Although all reported sporotrichosis cases in both the Brazil and Paraguay sides of the border were included, most isolates originated from Brazil. The population numbers for the cities at the triple border region are similar; the 2024 population of Foz do Iguaçu was 295,000 (*28*) and of Ciudad del Este was 339,000 (*29*). The numbers for the feline populations are not known for either city. The differences in sporotrichosis cases are partly the result of the river between the 2 countries, which halted spread originating from the east of Brazil. In addition, differences between the healthcare systems of the 2 countries might play a role. In Brazil, the Health Unic System (SUS) is a decentralized system, meaning that the city decides how the resources from the state and the federal government will be used (*30*). In Paraguay, a centralized health system has most action concentrated in the capital, Asunción, which is 324 km from the border (*30*). Although the Epidemiologic Laboratory in Ciudad del Este can track cases and diagnose sporotrichosis in cats and humans free of charge, not enough clinicians and veterinarians are available.

In this study, uncastrated and unvaccinated male cats represented most patients with feline sporotrichosis, as previously described (*31*). Unvaccinated cats may have comorbidities such as feline leukemia virus, calicivirus, herpes, and panleukopenia, leading to immunosuppression and rapid evolution to the disseminated form of sporotrichosis (*32*). Castration of male cats reduces production of testosterone and curbs behaviors of territory disputes and sexual intercourse between male and female cats, both of which usually involves fights with injuries, so reducing those interactions decreases chances of transmission to female cats and newborn kittens (*33*). Because most cats in this study were >12 months of age, public interventions (vaccination and neutering programs) at <12 months of age would likely reduce the risk for transmission of sporotrichosis. Such measures are crucial to control and prevent sporotrichosis based on a One Health approach, which also reinforces the need for public health education, especially about responsible cat ownership. Furthermore, the lack of awareness about this disease among health professionals is a primary difficulty in identifying sporotrichosis in humans and animals, making searching for cases more difficult (*12*). Thus, public health education on responsible feline ownership and increasing disease awareness in health professionals are the first steps toward preventing sporotrichosis outbreaks and providing effective treatment (*34*).

To show the genetic relatedness among the *S. brasiliensis* isolates, we performed short tandem repeat analysis (*35*). All isolates clustered in the Rio de Janeiro clade and were closely related to isolates from the south and southeastern parts of Brazil. Curitiba and other cities in the Brazil state of Paraná have steadily reported *S. brasiliensis* cases since 2011, and those isolates display identical or highly related genotypes (*16*). The introduction of *S. brasiliensis* in regions could happen by the movement of sick or colonized cats (*11*). The isolates from this study were closely related to those from Curitiba, which, like Foz do Iguaçu, is in Paraná state, although the 2 regions are >600 km apart. Even though all our isolates clustered in the Rio de Janeiro clade (*16*), we identified different genotypes, so multiple introductions cannot be excluded. Whole-genome sequencing is needed to elucidate the origin of *S. brasiliensis* in this region and whether all isolates originate from the same strain. Finally, we observed different clinical manifestations and mortality rates, but those differences were not related to different genetic backgrounds of isolates.

We determined MIC values for 9 different antifungal drugs for *S. brasiliensis* isolates in both the pure yeast and mycelial phases and microscopically confirmed results. According to the interpretation of an international multicenter study for definition of tentative ECVs for mycelial *S. brasiliensis* (*23*), all isolates were susceptible to amphotericin B, itraconazole, voriconazole, posaconazole, and terbinafine (*36*). Espinel-Ingroff et al. used standard incubation of 2–3 days at 35°C, according to the CLSI reference standard M38 for filamentous fungi, whereas in our study, we used 30°C to avoid conversion of the mold form. We found that incubation at 35°C induces transition to the yeast phase, taking up to 2 weeks for full transition. Because Espinel-Ingroff et al. did not perform microscopy, the ECVs possibly were established on a mixture of filamentous and yeast phases in that study. Moreover, many centers were excluded because of insufficient or unsuitable data, suggesting suboptimal methodology or implementation (*23*). Thus, additional studies are required to analyze the impact of yeast–mold mixtures resulting from short incubation at 35°C versus pure mold at 30°C on the MICs and to establish the ECVs. Nonetheless, the normal distribution of our MIC values indeed suggests an absence of non–wild-type isolates. Of note, we found mycelial phase MICs were overall higher than those for the yeast phase. One explanation for that difference is the higher concentration of melanin in the cell wall in the filamentous phase. Melanin is associated with a reduced susceptibility to antifungal drugs. However, it is important to note that MICs for most of the drugs in the mycelial phase were read at 100% inhibition compared with growth control, whereas at the yeast phase, inhibition was 50%. Therefore, direct comparisons of the MICs between both phases should be made with caution.

AFST results might not reflect in vivo treatment in the absence of clinical breakpoints (*34*). Nonetheless, for itraconazole, which is the first-choice drug for feline treatment (*15*,*34*), MICs of all isolates in both phases were below the tentative ECV, and similar results were reported earlier (*17*,*37*). In contrast, other studies recently found MICs of itraconazole and other azoles above the tentative ECV (*19*,*38*). Of note, the reported bimodal distribution with low and elevated MICs for itraconazole, and the identification of *cyp51* mutants (*19*) suggests that those MICs would also be well above the tentative ECV in conditions of pure mycelial and yeast phase (*23*). That discrepancy with our study might be because we included different strains. Our collection consisted of closely related genotypes only, and strains were isolated before start of treatment. Smaller MIC differences could also be explained by differences in AFST protocols, including the mixed presence of filamentous and yeast phases. Other factors, including panel preparation, media or reagents, and inoculum preparation, might influence AFST results. Finally, because the mycelial form had the least variation in our genotypically similar isolates, is easiest to use, and mycelial AFST data seemingly correspond to in vivo failure of voriconazole (*39*), the mycelial phase might be most suitable for AFST. A multicenter evaluation comparing robust AFST methodologies in a genotypically variable cohort would be needed to establish the best method to determine antifungal susceptibility for *S. brasiliensis*. Moreover, further research is needed to determine whether inoculum incubation at 35°C, in adherence with the M38 guideline for the mycelial phase, has influence on MIC outcome in comparison to incubation at 30°C.

We observed a high (61%) mortality rate in cats with the disseminated form and no sporothrichosis-related deaths in cats with the fixed cutaneous form. Other studies reported unfavorable clinical outcomes in 32%–59% of cats with the disseminated form (*40*–*42*). In our study, all cats with extracutaneous form achieved clinical cure, in contrast with previous studies, which generally considered that form to be indicative of a poor prognosis and high chances of treatment failure and death (*15*,*41*). Of note, all isolates in our study were genetically similar and displayed initially low MICs of itraconazole, used for sporothrichosis treatment in all cats. The 50% MIC levels of strains isolated from cats with the disseminated form that were cured were also not different from those that were not cured. Thus, the mortality rate in cats with the disseminated form is not because of initial elevated MICs, although we cannot exclude the development of reduced susceptibility overtime because we did not collect isolates after itraconazole treatment. The observation that transmission only involved itraconazole-susceptible isolates, because we did not find an isolate with high MIC in any cat, suggests that an increase in MICs was probably uncommon, if present at all. Moreover, some cats from the same household became infected months after each other, and in those households no elevated MICs were found, suggesting no resistance was acquired within the households despite itraconazole exposure. However, isolates collected after itraconazole exposure should be tested to formally exclude resistance development. Regarding treatment failure, other factors, such as disease progression, treatment variations, and host factors, likely were involved in treatment failure in this cohort. Besides clinical outcomes, treatment duration was different between clinical forms. Cats with the fixed cutaneous form were treated for fewer months. Disease progression is likely to play an important role, but erratic itraconazole pharmacokinetics might also be involved. 

For feline sporotrichosis, the proposed itraconazole dose by the guideline for the management of feline sporotrichosis caused by *S. brasiliensis* is 100 mg/24 hours for cats >3 kg (*15*). To our knowledge, however, a robust dose-response study evaluating the efficacy of that dose is lacking. When administering the medication, guidelines recommend opening the capsules over a small amount of wet food (*15*); however, no studies have verified the absorption degree of itraconazole administered that way. In disseminated cases, whether itraconazole can reach the mucous membranes at an adequate level for cure also is unknown. Moreover, the disease in the disseminated form could be too advanced to treat with itraconazole. However, suboptimal itraconazole blood levels prolong treatment and increase risk for resistance development in other diseases (*43*,*44*). Optimal dosing to reach effective serum itraconazole concentrations in severe disease would enable the best standard of care, but that information is not available for cats. Alternative therapeutic strategies should be investigated for the disseminated feline form to reduce mortality. 

In summary, our investigation of cat-transmitted sporotrichosis caused by *S. brasiliensis* at the triple border region of Brazil, Paraguay, and Argentina found that varying clinical manifestations of sporotrichosis and large differences in mortality rates were not caused by elevated itraconazole MICs. Early diagnosis and effective treatment for this infection are crucial to prevent disease progression, death, and transmission to other humans and animals.

Appendix Additional information for epidemiology of *Sporothrix brasiliensis* treatment failure without initial elevated itraconazole MICs in felids at border of Brazil.
