# Supplementary material for: Sporothrix brasiliensis Treatment Failure without Initial Elevated Itraconazole MICs in Felids at Border of Brazil
Source: Emerg Infect Dis. 2025 Sep;31(9):1783–92. doi: 10.3201/eid3109.250156 (PMC12407209; doi:10.3201/eid3109.250156)
Supplement: Appendix — Additional information for epidemiology of Sporothrix brasiliensis treatment failure without initial elevated itraconazole MICs in felids at border of Brazil. [file 25-0156-Techapp-s1.pdf]

Article DOI: https://doi.org/10.3201/eid3109.250156

*EID cannot ensure accessibility for supplementary materials supplied by authors. Readers who have difficulty accessing supplementary content should contact the authors for assistance.*

# *Sporothrix brasiliensis* Treatment Failure without Initial Elevated Itraconazole MICs in Felids at Border of Brazil

## Appendix

### Additional Materials and Methods

For short tandem repeat (STR) genotyping, nine markers were amplified with three multiplex PCRs and subsequently analyzed with a 3500 XL genetic analyzer (Applied Biosystems, Foster City, CA, USA) (*1*). Copy numbers were determined using GeneMapper 5 (Applied Biosystems) and the genetic relatedness between the current and previously genotyped isolates was assessed with BioNumerics v7.6.1 (Applied Maths NV, Sint-Martems-Latem, Belgium).

For antifungal susceptibility testing (AFST) in the mycelial phase incubation was performed at 30°C and minimum inhibitory concentrations (MICs) were read after 72 hours instead of the usual 48 hours (*2*). For inoculum preparation, isolates were suspended in 0.9% NaCl with 0.05% Tween 20 and the transmission of the suspension was measured at 530 nm using Spectrophotometer IV Control Genesys 20. Transmission range of 80%–82% (0.9 – 5.0 × $10^6$ CFU/mL) was used. The suspension was diluted (1:50) in RPMI 1640 (0.2% glucose) with a final conidial concentration of 0.4 × $10^4$ to 5 × $10^4$ CFU/mL. *Aspergillus flavus* ATCC 204304 was used as quality control. Amphotericin B (Bristol Myers Squib, Woerden, The Netherlands), fluconazole (Merck, Darmstadt, Germany), itraconazole (Janssen Cilag, Breda, The Netherlands), voriconazole (Pfizer Central Research, New York, USA), posaconazole (Merck), isavuconazole (Basilea Pharmaceutica, Basel, Switzerland), terbinafine (VWR, Leicestershire, UK), anidulafungin (Merck) and micafungin (Astellas Pharma, Tokyo, Japan) were tested. For amphotericin B, itraconazole, voriconazole, posaconazole, and isavuconazole, the MIC endpoint was the lowest concentration that produced complete inhibition of growth. For fluconazole, the

MIC was defined as the lowest concentration at which there was ≤50% growth reduction as compared to growth control. For echinocandins, it was the lowest concentration producing a visual change in the appearance of the growth, defined as the minimum effective concentration (MEC). For terbinafine, the MIC was defined as the lowest concentration at which there was ≤80% growth reduction when compared to the growth control. For mycelial phase, tentative epidemiologic cutoff values (ECVs) at 97.5% as per CLSI M57 recommendation, according to Espinel-Ingroff were implemented for amphotericin B, itraconazole, voriconazole, posaconazole and terbinafine (*3*).

For inoculum preparation of AFST in the yeast phase, isolates were suspended in 0.9% NaCl equal to 0.5 McFarland. The transmission of the suspension was measured at 530 nm using Spectrophotometer IV Control Genesys 20. A transmission range of 75%–77% ($1.0 – 5.0 \times 10^6$ CFU/mL) was used. The suspension was diluted 100 times in RPMI 1640. *Candida krusei* ATCC 6258 and *Candida parapsilosis* ATCC 22019 were used as quality controls. The same antifungals as for the mycelial phase were tested. The MICs were read visually after 96-120 hours of incubation at 35°C. For amphotericin B, fluconazole and terbinafine, the MIC endpoint was identical as used for mycelial phase and for itraconazole, voriconazole, posaconazole, isavuconazole, anidulafungin and micafungin, the MIC was defined as the lowest concentration at which there was a ≤50% growth reduction.

**References**


1. Spruijtenburg B, Bombassaro A, Meijer EFJ, Rodrigues AM, Grisolia ME, Vicente VA, et al. *Sporothrix brasiliensis* genotyping reveals numerous independent zoonotic introductions in Brazil. J Infect. 2023;86:610–3. PubMed https://doi.org/10.1016/j.jinf.2023.02.034

2. Clinical and Laboratory Standards Institute. Reference method for broth dilution antifungal susceptibility testing of filamentous fungi. CLSI standard M38. 3rd edition. Wayne (PA): The Institute; 2017.

3. Espinel-Ingroff A, Abreu DPB, Almeida-Paes R, Brilhante RSN, Chakrabarti A, Chowdhary A, et al. Multicenter, international study of MIC/MEC distributions for definition of epidemiological cutoff values for *Sporothrix* species identified by molecular methods. Antimicrob Agents Chemother. 2017;61:e01057–17. PubMed https://doi.org/10.1128/AAC.01057-17

**Appendix Table 1.** Accession numbers (NCBI), date of sampling and geographic data of the animals with proven sporotrichosis in the triple border region*

| CMRP | Accession no. | Date | Neighborhood | City | Country |
|---|---|---|---|---|---|
| CMRP6060 | PQ741666 | 07/07/2021 | Jardim Alice I | Foz do Iguaçu | Brazil |
| CMRP5585 | PQ741608 | 09/07/2021 | Parque Residencial Morumbi II | Foz do Iguaçu | Brazil |
| CMRP6061 | PQ741667 | 09/07/2021 | Parque Residencial Morumbi II | Foz do Iguaçu | Brazil |
| CMRP6062 | PQ741668 | 09/07/2021 | Jardim Copacabana | Foz do Iguaçu | Brazil |
| CMRP5586 | PQ741609 | 30/07/2021 | Parque Residencial Morumbi | Foz do Iguaçu | Brazil |
| CMRP6063 | PQ741669 | 30/07/2021 | Parque Residencial Morumbi | Foz do Iguaçu | Brazil |
| CMRP6064 | PQ741670 | 09/08/2021 | Portal da Foz | Foz do Iguaçu | Brazil |
| CMRP6065 | PQ741671 | 09/08/2021 | Portal da Foz | Foz do Iguaçu | Brazil |
| CMRP6066 | PQ741672 | 16/08/2021 | Portal da Foz | Foz do Iguaçu | Brazil |
| CMRP6067 | PQ741673 | 16/08/2021 | Portal da Foz | Foz do Iguaçu | Brazil |
| CMRP6068 | PQ741674 | 16/08/2021 | Portal da Foz | Foz do Iguaçu | Brazil |
| CMRP6069 | PQ741675 | 16/09/2021 | Parque Residencial Santa Rita | Foz do Iguaçu | Brazil |
| CMRP5587 | PQ741610 | 16/09/2021 | Jardim Alice I | Foz do Iguaçu | Brazil |
| CMRP6070 | PQ741676 | 24/09/2021 | Jardim Europa | Foz do Iguaçu | Brazil |
| CMRP6071 | PQ741677 | 24/09/2021 | Parque Residencial Morumbi II | Foz do Iguaçu | Brazil |
| CMRP6072 | PQ741678 | 24/09/2021 | Parque Residencial Morumbi II | Foz do Iguaçu | Brazil |
| CMRP6073 | PQ741679 | 24/09/2021 | Parque Residencial Morumbi | Foz do Iguaçu | Brazil |
| CMRP5588 | PQ741611 | 29/12/2021 | Beverli Falls Park | Foz do Iguaçu | Brazil |
| CMRP5589 | PQ741612 | 03/01/2022 | Jardim das Flores | Foz do Iguaçu | Brazil |
| CMRP5590 | PQ741613 | 18/01/2022 | Jardim das Flores | Foz do Iguaçu | Brazil |
| CMRP5591 | PQ741614 | 18/01/2022 | Jardim das Flores | Foz do Iguaçu | Brazil |
| CMRP5592 | PQ741615 | 01/02/2022 | Jardim Curitibano | Foz do Iguaçu | Brazil |
| CMRP5593 | PQ741616 | 04/02/2022 | Conjunto C | Foz do Iguaçu | Brazil |
| CMRP5594 | PQ741617 | 04/02/2022 | Conjunto C | Foz do Iguaçu | Brazil |
| CMRP6074 | PQ741680 | 10/03/2022 | Parque Residencial Morumbi II | Foz do Iguaçu | Brazil |
| CMRP6075 | PQ741681 | 16/03/2022 | Jardim Nacional | Foz do Iguaçu | Brazil |
| CMRP6077 | PQ741682 | 05/04/2022 | Cidade Nova II | Foz do Iguaçu | Brazil |
| CMRP6078 | PQ741683 | 19/04/2022 | Conjunto C | Foz do Iguaçu | Brazil |
| CMRP6079 | PQ741684 | 19/04/2022 | Cidade Nova II | Foz do Iguaçu | Brazil |
| CMRP6080 | PQ741685 | 27/04/2022 | Parque Residencial Santa Rita | Foz do Iguaçu | Brazil |
| CMRP6082 | PQ741686 | 02/06/2022 | Conjunto C | Foz do Iguaçu | Brazil |
| CMRP6083 | PQ741687 | 02/06/2022 | Parque Residencial Morumbi II | Foz do Iguaçu | Brazil |
| CMRP6084 | PQ741688 | 07/06/2022 | Residencial Morumbi III | Foz do Iguaçu | Brazil |
| CMRP5785 | PQ741618 | 07/06/2022 | Parque Residencial Italia | Foz do Iguaçu | Brazil |
| CMRP6085 | PQ741689 | 08/06/2022 | Vila São Sebastião | Foz do Iguaçu | Brazil |
| CMRP6086 | PQ741690 | 14/06/2022 | Vila Borges | Foz do Iguaçu | Brazil |
| CMRP6087 | PQ741691 | 14/06/2022 | Vila Borges | Foz do Iguaçu | Brazil |
| CMRP6088 | PQ741692 | 20/06/2022 | Jardim São Paulo II | Foz do Iguaçu | Brazil |
| CMRP6089 | PQ741693 | 21/06/2022 | Jardim São Paulo II | Foz do Iguaçu | Brazil |
| CMRP6090 | PQ741694 | 21/06/2022 | Porto Belo | Foz do Iguaçu | Brazil |
| CMRP5786 | OR501573 | 22/06/2022 | NA | Ciudad del Este | Paraguay |
| CMRP6091 | PQ741695 | 07/07/2022 | Portal da Foz | Foz do Iguaçu | Brazil |
| CMRP6092 | PQ741696 | 07/07/2022 | Portal da Foz | Foz do Iguaçu | Brazil |
| CMRP6093 | PQ741697 | 14/07/2022 | Portal da Foz | Foz do Iguaçu | Brazil |
| CMRP6094 | PQ741698 | 14/07/2022 | Portal da Foz | Foz do Iguaçu | Brazil |
| CMRP6095 | PQ741699 | 28/07/2022 | Parque Residencial Morumbi II | Foz do Iguaçu | Brazil |
| CMRP6096 | PQ741700 | 11/08/2022 | Vila Borges | Foz do Iguaçu | Brazil |
| CMRP6097 | PQ741701 | 11/08/2022 | Parque Residencial Morumbi II | Foz do Iguaçu | Brazil |
| CMRP6098 | PQ741702 | 19/08/2022 | Vila São Sebastião | Foz do Iguaçu | Brazil |
| CMRP5787 | OR501574 | 19/08/2022 | NA | Ciudad del Este | Paraguay |
| CMRP6099 | PQ741703 | 24/08/2022 | Conjunto C | Foz do Iguaçu | Brazil |
| CMRP6100 | PQ741704 | 26/08/2022 | Conjunto C | Foz do Iguaçu | Brazil |
| CMRP6101 | PQ741705 | 26/08/2022 | Conjunto C | Foz do Iguaçu | Brazil |
| CMRP6102 | PQ741706 | 26/08/2022 | Conjunto C | Foz do Iguaçu | Brazil |
| CMRP6103 | PQ741707 | 06/09/2022 | Jardim Residencial São Roque | Foz do Iguaçu | Brazil |
| CMRP6104 | PQ741708 | 09/09/2022 | Parque Residencial Morumbi II | Foz do Iguaçu | Brazil |
| CMRP6105 | PQ741709 | 20/09/2022 | Profilurb I | Foz do Iguaçu | Brazil |
| CMRP6106 | PQ741710 | 05/10/2022 | Jardim Bela Vista de Itaipu III | Foz do Iguaçu | Brazil |
| CMRP6107 | PQ741711 | 05/10/2022 | Jardim Oriente | Foz do Iguaçu | Brazil |
| CMRP6108 | PQ741712 | 05/10/2022 | Profilurb I | Foz do Iguaçu | Brazil |
| CMRP5791 | PQ741619 | 30/11/2022 | NA | Hernandarias | Paraguay |
| CMRP5792 | PQ741620 | 10/12/2022 | NA | Hernandarias | Paraguay |
| CMRP5915 | PQ741621 | 19/01/2023 | NA | Hernandarias | Paraguay |
| CMRP5916 | PQ741622 | 19/01/2023 | NA | Hernandarias | Paraguay |
| CMRP5917 | PQ741623 | 12/05/2023 | Portal da Foz | Foz do Iguaçu | Brazil |
| CMRP5918 | PQ741624 | 12/05/2023 | Portal da Foz | Foz do Iguaçu | Brazil |
| CMRP5919 | PQ741625 | 25/05/2023 | Portal da Foz | Foz do Iguaçu | Brazil |

| CMRP | Accession no. | Date | Neighborhood | City | Country |
|---|---|---|---|---|---|
| CMRP5940 | PQ741640 | 25/05/2023 | Parque Residencial Morumbi II | Foz do Iguaçu | Brazil |
| CMRP5941 | PQ741641 | 25/05/2023 | Parque Residencial Morumbi II | Foz do Iguaçu | Brazil |
| CMRP5921 | PQ741626 | 01/06/2023 | Vila Guarani | Foz do Iguaçu | Brazil |
| CMRP5922 | PQ741627 | 08/06/2023 | NA | Ciudad del Este | Paraguay |
| CMRP5923 | PQ741628 | 12/06/2023 | Jardim Novo Horizonte | Foz do Iguaçu | Brazil |
| CMRP5924 | PQ741629 | 12/06/2023 | Jardim Novo Horizonte | Foz do Iguaçu | Brazil |
| CMRP5925 | PQ741630 | 22/06/2023 | Centro | Foz do Iguaçu | Brazil |
| CMRP5942 | PQ741642 | 22/06/2023 | Profilurb I | Foz do Iguaçu | Brazil |
| CMRP5926 | PQ741631 | 28/06/2023 | Jardim São Luiz | Foz do Iguaçu | Brazil |
| CMRP5927 | PQ741632 | 28/06/2023 | Jardim São Luiz | Foz do Iguaçu | Brazil |
| CMRP5943 | PQ741643 | 30/06/2023 | Vila A | Foz do Iguaçu | Brazil |
| CMRP5944 | PQ741644 | 30/06/2023 | Jd. Califórnia II | Foz do Iguaçu | Brazil |
| CMRP5928 | PQ741633 | 06/07/2023 | Jardim Guaira | Foz do Iguaçu | Brazil |
| CMRP5945 | PQ741645 | 06/07/2023 | Bubas | Foz do Iguaçu | Brazil |
| CMRP5946 | PQ741646 | 11/07/2023 | Profilurb I | Foz do Iguaçu | Brazil |
| CMRP5929 | PQ741634 | 11/07/2023 | Cohapar III | Foz do Iguaçu | Brazil |
| CMRP5947 | PQ741647 | 13/07/2023 | Conjunto C | Foz do Iguaçu | Brazil |
| CMRP5948 | PQ741648 | 13/07/2023 | Loteamento Bela Vista de Itaipu II | Foz do Iguaçu | Brazil |
| CMRP5930 | PQ741635 | 13/07/2023 | Jardim Cedro | Foz do Iguaçu | Brazil |
| CMRP5931 | PQ741636 | 20/07/2023 | Conjunto C | Foz do Iguaçu | Brazil |
| CMRP5949 | PQ741649 | 20/07/2023 | Portal da Foz | Foz do Iguaçu | Brazil |
| CMRP5932 | PQ741637 | 26/07/2023 | Jardim Duarte | Foz do Iguaçu | Brazil |
| CMRP5950 | PQ741650 | 28/07/2023 | Jardim Novo Horizonte | Foz do Iguaçu | Brazil |
| CMRP5933 | PQ741638 | 28/07/2023 | Jardim Novo Horizonte | Foz do Iguaçu | Brazil |
| CMRP5935 | PQ741639 | 28/07/2023 | Jardim Eliza II | Foz do Iguaçu | Brazil |
| CMRP5952 | PQ741651 | 16/08/2023 | Jardim California | Foz do Iguaçu | Brazil |
| CMRP5953 | PQ741652 | 16/08/2023 | Centro | Foz do Iguaçu | Brazil |
| CMRP5955 | PQ741653 | 19/08/2023 | NA | Ciudad del Este | Paraguay |
| CMRP5956 | PQ741654 | 24/08/2023 | Jardim das Flores | Foz do Iguaçu | Brazil |
| CMRP5957 | PQ741655 | 24/08/2023 | Parque Residencial Morumbi II | Foz do Iguaçu | Brazil |
| CMRP5958 | PQ741656 | 04/09/2023 | Jardim São Paulo II | Foz do Iguaçu | Brazil |
| CMRP5959 | PQ741657 | 04/09/2023 | Jardim São Paulo II | Foz do Iguaçu | Brazil |
| CMRP5960 | PQ741658 | 04/09/2023 | Conjunto Habitacional Fernanda | Foz do Iguaçu | Brazil |
| CMRP5961 | PQ741659 | 06/09/2023 | Jardim Tropical | Foz do Iguaçu | Brazil |
| CMRP5962 | PQ741660 | 06/09/2023 | Parque Ouro Verde | Foz do Iguaçu | Brazil |
| CMRP5963 | PQ741661 | 12/09/2023 | Vila Guarani | Foz do Iguaçu | Brazil |
| CMRP5964 | PQ741662 | 12/09/2023 | Parque Residencial Morumbi III | Foz do Iguaçu | Brazil |
| CMRP5965 | PQ741663 | 13/09/2023 | Cohapar III | Foz do Iguaçu | Brazil |
| CMRP5966 | PQ741664 | 15/09/2023 | Loteamento Bela Vista de Itaipu II | Foz do Iguaçu | Brazil |
| CMRP5967 | PQ741665 | 15/09/2023 | Loteamento Bela Vista de Itaipu II | Foz do Iguaçu | Brazil |
| CMRP6111 | PQ741713 | 25/10/2023 | Jardim São Luiz | Foz do Iguaçu | Brazil |

*NA, not applicable.

**Appendix Table 2.** Comparison between clinical outcome and geometric mean MIC*

| | Geometric mean MIC | | | | | | | | | | | | | | | | | |
|---|---|---|---|---|---|---|---|---|---|---|---|---|---|---|---|---|---|---|
| | AMB | | FLU | | ITC | | VOR | | POS | | ISA | | TRB | | AFG | | MCF | |
| Outcome | M | Y | M | Y | M | Y | M | Y | M | Y | M | Y | M | Y | M | Y | M | Y |
| Cutaneous disseminated disease | | | | | | | | | | | | | | | | | | |
| Death | 1 | 0.7 | 64 | 20 | 0.3 | 0.05 | 15 | 0.3 | 0.4 | 0.05 | 8 | 0.07 | 0.09 | 0.14 | 0.008 | 0.05 | 0.008 | 0.04 |
| Clinical cure | 1 | 0.8 | 64 | 21 | 0.4 | 0.04 | 16 | 0.4 | 0.4 | 0.06 | 8 | 0.06 | 0.08 | 0.13 | 0.008 | 0.07 | 0.008 | 0.06 |
| Lost to follow up | 1 | 0.7 | 64 | 25 | 0.5 | 0.09 | 16 | 0.6 | 0.5 | 0.12 | 8 | 0.19 | 0.07 | 0.07 | 0.008 | 0.07 | 0.008 | 0.06 |
| Euthanized | 1 | 0.5 | 64 | 28 | 0.3 | 0.03 | 14 | 0.5 | 0.3 | 0.04 | 5 | 0.06 | 0.08 | 0.04 | 0.008 | 0.05 | 0.008 | 0.12 |
| Fixed cutaneous disease | | | | | | | | | | | | | | | | | | |
| Clinical cure | 1 | 0.6 | 64 | 15 | 0.3 | 0.04 | 15 | 0.2 | 0.4 | 0.05 | 7 | 0.05 | 0.08 | 0.14 | 0.008 | 0.06 | 0.008 | 0.06 |
| Lost to follow up | 1 | 0.4 | 64 | 32 | 0.28 | 0.07 | 12 | 0.3 | 0.3 | 0.06 | 6 | 0.08 | 0.14 | 0.12 | 0.01 | 0.09 | 0.01 | 0.05 |
| Euthanized | 1 | 1 | 64 | 25 | 0.3 | 0.02 | 16 | 0.3 | 0.5 | 0.03 | 6 | 0.02 | 0.09 | 0.12 | 0.01 | 0.03 | 0.008 | 0.02 |
| Extracutaneous disease | | | | | | | | | | | | | | | | | | |
| Clinical cure | 2 | 1 | 64 | 18 | 0.37 | 0.05 | 16 | 0.6 | 0.4 | 0.06 | 10 | 0.04 | 0.14 | 0.18 | 0.008 | 0.02 | 0.008 | 0.04 |

*AMB, amphotericin B; AFG, anidulafungin; FLU, fluconazole; ISA, isavuconazole; ITC, itraconazole; M, mycelial phase; MFG, micafungin; POS, posaconazole; TRB, terbinafine; VOR, voriconazole; Y, yeast phase.

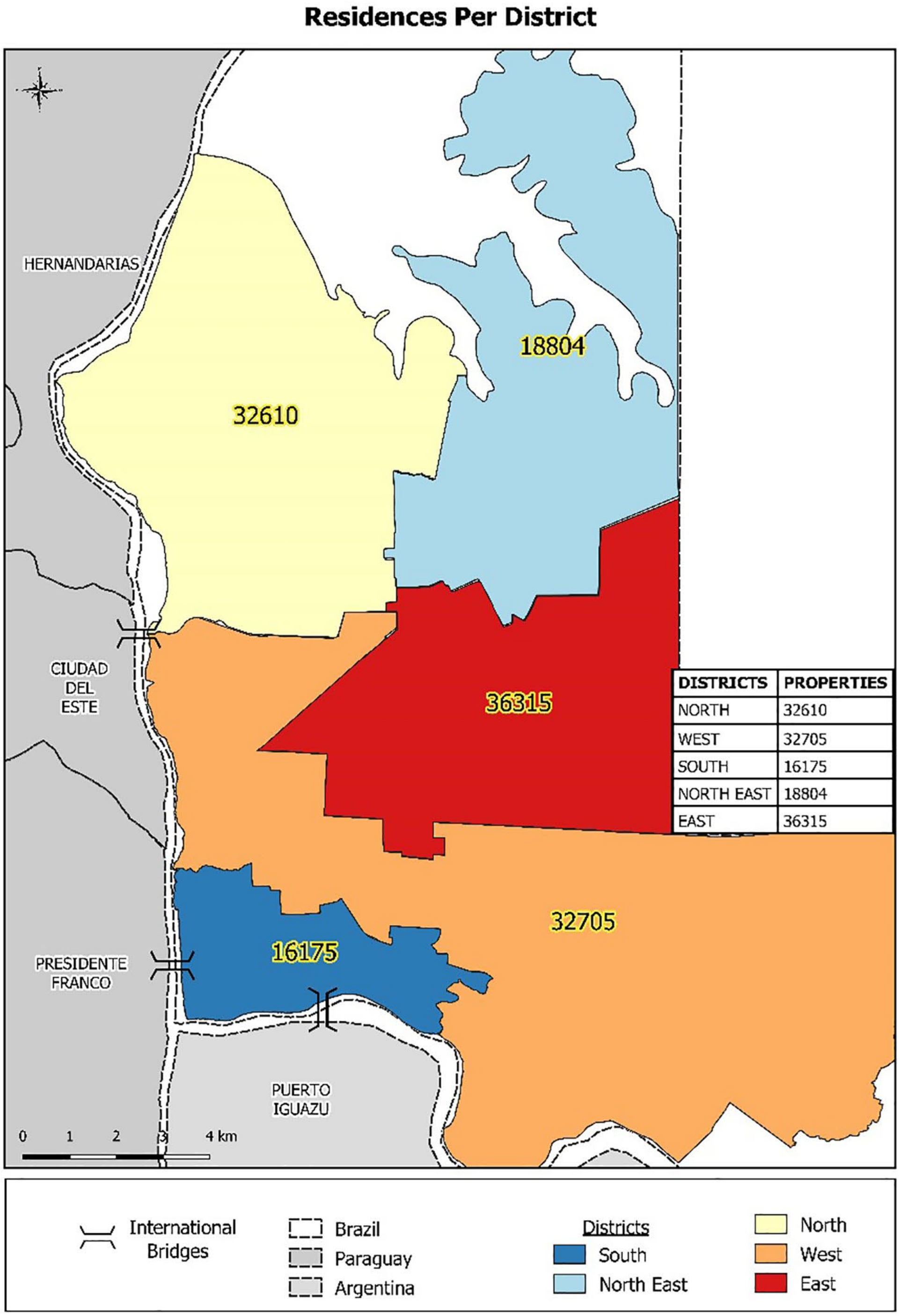


**Appendix Figure 1.** Number of residences per district of Foz do Iguaçu, Brazil.

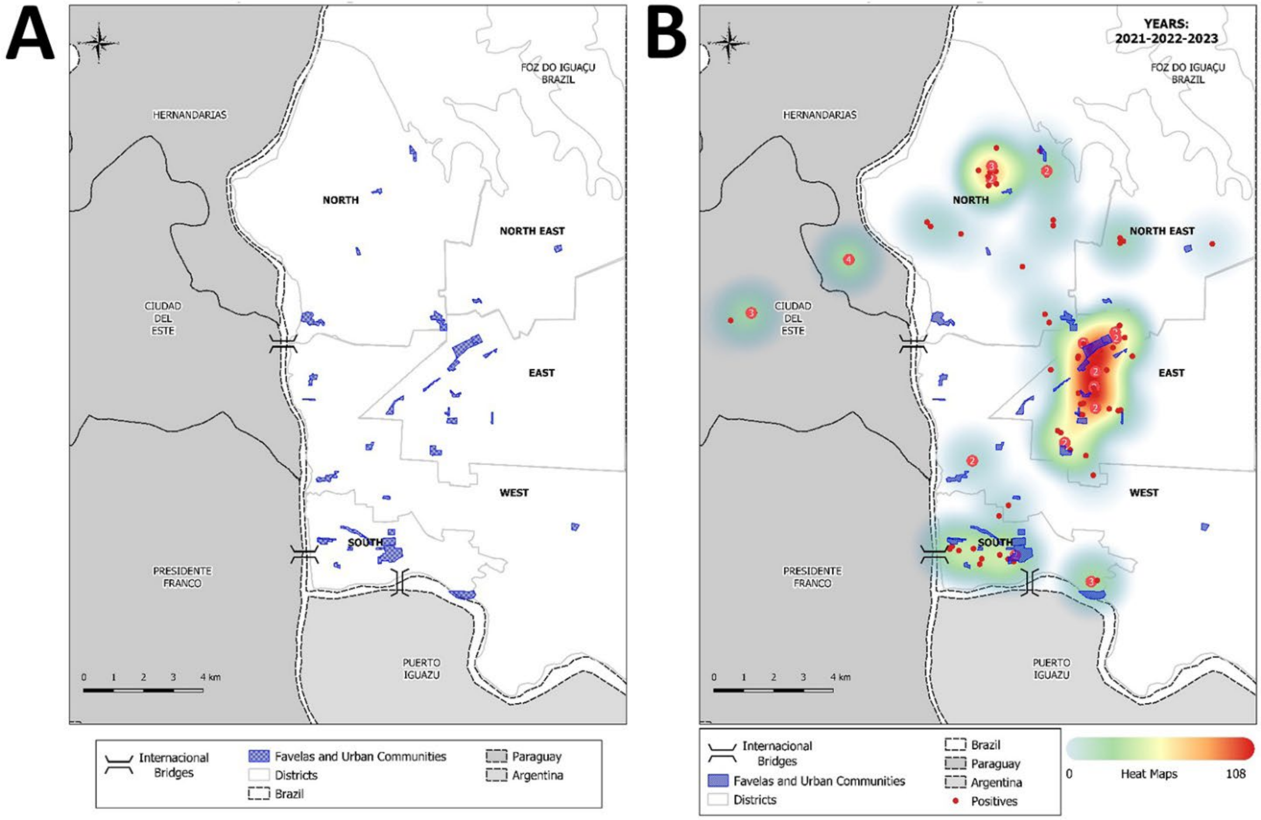


**Appendix Figure 2.** Location of the favelas and poor urban communities (FPUC) in Foz do Iguaçu, Brazil (A). Map with FPUC + all sporotrichosis cases 2021-2023 (B).

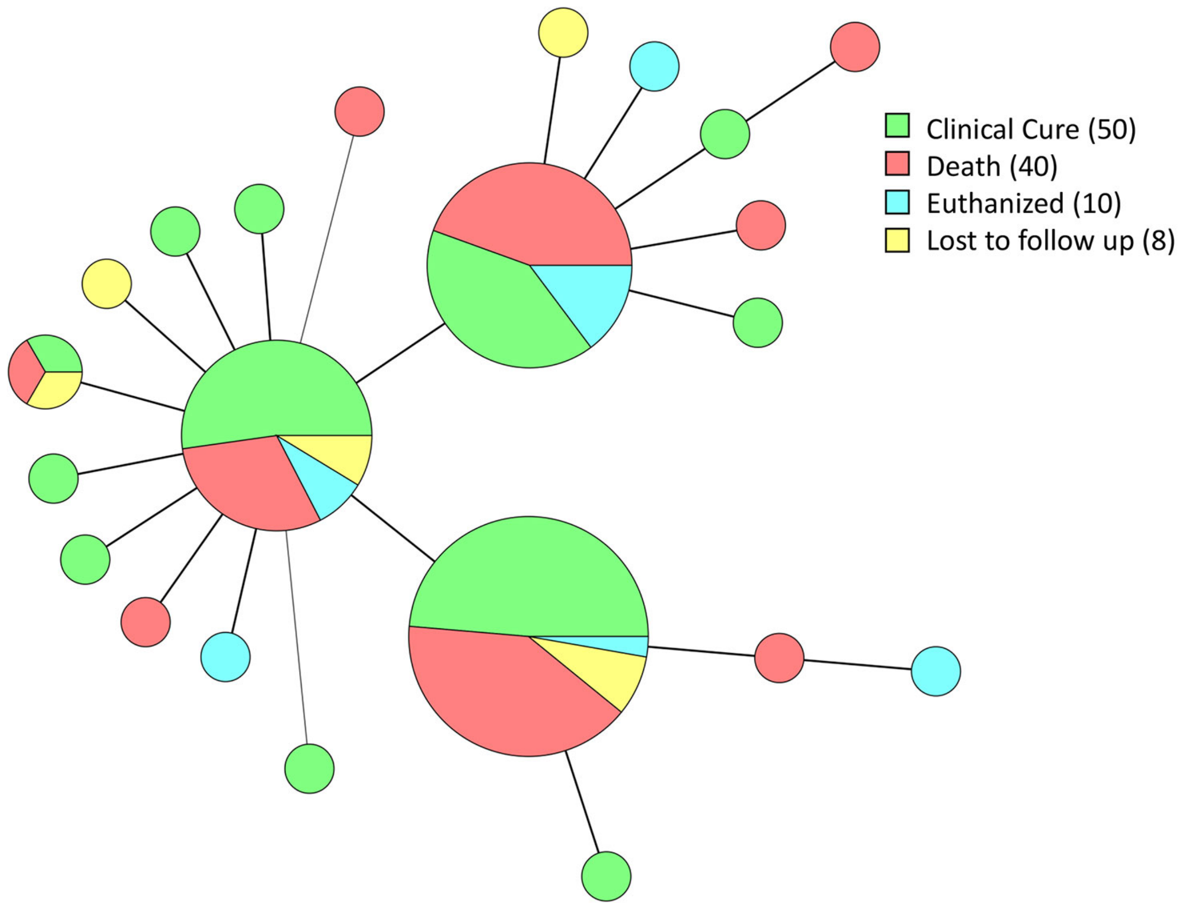


**Appendix Figure 3.** Minimum-spanning tree of 108 *Sporothrix brasiliensis* isolates based on nine microsatellite markers. Isolates are colored after the clinical outcome and branch lengths indicate the genetic relatedness, with thick solid lines (variation in one marker) and thin solid lines (variation in two markers).
